# Effects of needs-based patient education on self-efficacy and health outcomes in people with rheumatoid arthritis: a multicentre, single blind, randomised controlled trial

**DOI:** 10.1136/annrheumdis-2014-207171

**Published:** 2015-07-10

**Authors:** M Ndosi, D Johnson, T Young, B Hardware, J Hill, C Hale, J Maxwell, E Roussou, A Adebajo

**Affiliations:** 1 Leeds Institute of Rheumatic and Musculoskeletal Medicine, University of Leeds, Leeds, UK; 2 School of Healthcare, University of Leeds, Leeds, UK; 3 Department of Rheumatology, Barnsley Hospital NHS Foundation Trust, Barnsley, UK; 4 Research & Development Department, Barnsley Hospital NHS Foundation Trust, Barnsley, UK; 5 School of Health and Related Research, University of Sheffield, Sheffield, UK; 6 Department of Rheumatology, Rotherham Hospital NHS Foundation Trust, Rotherham, UK; 7 Department of Rheumatology, King George Hospital, Barking Havering and Redbridge University Hospitals NHS Trust, Ilford, UK

**Keywords:** Health services research, Rheumatoid Arthritis, Rehabilitation, Multidisciplinary team-care, Patient perspective

## Abstract

**Objectives:**

The Educational Needs Assessment Tool (ENAT) is a self-completed questionnaire, which allows patients with arthritis to prioritise their educational needs. The aim of this study was to evaluate the effects of needs-based patient education on self-efficacy, health outcomes and patient knowledge in people with rheumatoid arthritis (RA).

**Methods:**

Patients with RA were enrolled into this multicentre, single-blind, parallel-group, pragmatic randomised controlled trial. Patients were randomised to either the intervention group (IG) where patients completed ENAT, responses of which were used by the clinical nurse specialist to guide patient education; or control group (CG) in which they received patient education without the use of ENAT. Patients were seen at weeks 0, 16 and 32. The primary outcome was self-efficacy (Arthritis Self Efficacy Scale (ASES)-Pain and ASES-Other symptoms). Secondary outcomes were health status (short form of Arthritis Impact Measurement Scale 2, AIMS2-SF) and patient knowledge questionnaire-RA. We investigated between-group differences using analysis of covariance, adjusting for baseline variables.

**Results:**

A total of 132 patients were recruited (IG=70 and CG=62). Their mean (SD) age was 54 (12.3) years, 56 (13.3)  years and disease duration 5.2 (4.9) years, 6.7 (8.9) years for IG and CG, respectively. There were significant between-group differences, in favour of IG at week 32 in the primary outcomes, ASES-Pain, mean difference (95% CI) −4.36 (1.17 to 7.55), t=−2.72, p=0.008 and ASES-Other symptoms, mean difference (95% CI) −5.84 (2.07 to 9.62), t=−3.07, p=0.003. In secondary outcomes, the between-group differences favoured IG in AIMS2-SF Symptoms and AIMS2-SF Affect. There were no between-group differences in other secondary outcomes.

**Conclusions:**

The results suggest that needs-based education helps improve patients’ self-efficacy and some aspects of health status.

**Trial registration number:**

ISRCTN51523281.

## Introduction

Rheumatoid arthritis (RA) is a systemic chronic inflammatory disease characterised by joint swelling, joint tenderness and destruction of synovial joints, potentially leading to severe disability and premature mortality.^1^ ^2^ RA has a negative impact on individuals’ physical and psychological functioning, which contributes significantly to the burden of disease.^2–4^ The primary goal of treatment is to suppress disease activity, thus preventing structural damage and optimising function and social participation.^5^

Patient education can be defined as planned organised learning experiences designed to support and enable people to manage life with their condition and optimise their health and well-being.^6^ Patient education has been recommended as an integral part of management in RA as this prepares the patient to undertake self-management activities and adhere to all their treatments.^6–8^ Patient education needs to aim at improving patients’ self-efficacy as this is a predictor of self-management and other health outcomes.^9–12^ Furthermore, there is now an understanding that effective management of chronic conditions must be based on shared decision-making between the patient and healthcare providers,^10^ ^13^ the principle which underlies the current RA treatment recommendations.^5^ ^14^ ^15^

In rheumatology services, provision of patient education is considered to be an important role of the rheumatology nurses^8^ ^16^ ^17^ and patients value the education received from them.^18–22^ This has been supported by nurse-led care effectiveness randomised controlled trials (RCTs)^21–27^ and European League against Rheumatism (EULAR) recommendations for the role of the nurse in inflammatory arthritis.^28^ Although people with RA value patient education, there may be a discrepancy between their perception of their educational needs and the perception of their providers.^29^ If the provided education does not match patients’ perceived educational needs, it may not be acted upon, which would be a waste of health service resource. The Educational Needs Assessment Tool (ENAT) is a self-completed questionnaire, which allows patients with arthritis to prioritise their educational needs.^30^ The tool was developed with patients and it has 39 items which are grouped into seven domains: managing pain, movement, feelings, arthritis process, treatments, self-help measures and support. ENAT has been validated as a generic instrument for rheumatic diseases across nine European countries.^31–33^ It can be used as a clinical tool to guide patient education or as an outcome measure in research. Completion of ENAT just before the clinical consultation enables the health professional to provide education based on the patient's immediate priorities.

Recently, EULAR evidence-based recommendations for patient education in inflammatory arthritis have been published^6^ and they have recommended that content and delivery of patient education should be individually tailored and needs-based. Recent systematic reviews^6^ ^11^ ^34^ and RCTs^12^ ^21^ ^22^ have shown that tailored patient education is likely to improve health outcomes including self-efficacy. Two of the RCTs^21^ ^22^ used ENAT as an outcome measure but not as a clinical tool to guide individual patient education. The present study is the first RCT to use ENAT as part of the intervention. The aim of this study was to evaluate the effects of needs-based patient education on self-efficacy, patient knowledge and health status (physical function, symptoms, role/work, social interaction and psychological status/affect).

## Methods

### Study design

This was a multicentre, single-blind, parallel-group, pragmatic RCT conducted between April 2010 and August 2013. The trial comprised two groups: (1) The need-based patient education (the intervention group, IG) and (2) patient education given in the usual way, without the use of ENAT (the control group, CG). The study was conducted in accordance with Good Clinical Practice in research and the Research Governance Framework for Health and Social Care.^35^ All patients gave their written consent after having received full information about the study objectives and content. This trial was registered (ISRCTN51523281) and the protocol has been published elsewhere.^36^

### Participants and randomisation

Patients were identified from the lists of new clinic referrals at six rheumatology centres in Yorkshire, Shropshire, Coventry, Devon and London, UK. The inclusion criteria were: a positive diagnosis of RA done by a rheumatologist, using the 1987 American College of Rheumatology (ACR) criteria^37^ or the 2010 ACR/EULAR criteria^38^ and age 18 years or older. The exclusion criteria were: suffering from a severe mental health problem and inability to complete questionnaire unaided. Patients who fulfilled the inclusion criteria were sent a study invitation letter with patient information sheet by post. At each centre, patients were approached by a recruiter (a research nurse or a research associate) who was not involved in giving study interventions. Those who agreed to participate were asked to attend their usual clinic appointment and their written consent was sought at this visit. Following informed consent, patients were randomised on a 1:1 basis to either the needs-based education group or the usual care group. The randomisation was by block randomisation done by computer generated random numbers. The results of the randomisation were held at each centre in sealed in brown envelopes.

### Blinding

The patients (in CG and IG) were blinded to the nature of the intervention. In the patient information leaflet, all patients were informed about the purpose of the study and about completing ‘some’ questionnaires prior to their clinic consultation. During the clinic visit, patients were seen at separate appointment times and they were not told which group they were in. Although both groups completed a set of questionnaires and were given patient education, they did not know that IG additionally completed ENAT as part of their questionnaire set and that their responses were used to guide patient education. The clinical nurse specialists (CNS) who provided the patient education were not blinded.

### Interventions

Rheumatology CNS saw all patients (CG and IG) in the clinic on three occasions, at weeks 0, 16 and 32. These time points relate to the normal follow-up practice. As a part of the usual care, the CNS monitored disease activity, other symptoms and patient coping, adherence to treatments and side effects. Other activities included making referrals to other health professionals, providing psychosocial support and giving patient education without the use of ENAT. Consultations in IG were similar to CG except that patients completed ENAT at each clinic visit, prior to their consultation with the CNS and their responses were used to direct patient education at that time. After each consultation in both groups, the CNS completed a form to record the patient education which has been provided.

### Outcome measures

The primary outcome was perceived self-efficacy, measured by the short form of the Arthritis Self Efficacy Scale (ASES)^39^ at week 32. The questionnaire has been validated in the UK and it has two subscales ASES-Pain and ASES-Other symptoms with score ranges 5–50 and 6–60, respectively, higher scores reflecting higher self-efficacy. Secondary outcomes were: (1) disease-specific health status assessed by the short form of Arthritis Impact Measurement Scale 2 (AIMS2-SF),^40^ which measures physical function, symptoms (pain, stiffness and sleep), role, social interaction and psychological status. AIMS2-SF is a valid and reliable measure in RA and each subscale score ranges from 0 to 10, 0 being good health status,^40^ and (2) patient knowledge of their disease, measured by Patient Knowledge Questionnaire (PKQ-RA).^41^ PKQ-RA has been shown to be a valid, reliable and sensitive tool for measuring the acquisition of RA knowledge following educational intervention.^41^ The score ranges from 0 to 12, higher scores indicating higher level of knowledge. All outcomes were measured at weeks 0, 16 and 32. Attempts were made to follow-up all randomised participants with incomplete data, by telephone and letters with follow-up questionnaires. Additionally, although in this study, ENAT was used as a clinical tool and not as an outcome measure, we analysed ENAT scores of IG aiming to assess possible trends in patients’ educational needs over time.

### Sample size calculation

The sample size calculation was based on the clinically meaningful change in the ASES (ASES-Pain and ASES-Other symptoms) scores.^39^ Assuming a mean difference of 5.5 (SD=10.0) as being clinically meaningful for the ASES-Pain and ASES-Other symptoms scales with a 5% significance level and 80% power, 52 patients were needed per arm of the trial (total=104). To allow for a 25% dropout rate, 130 patients were recruited.

### Data analysis

With the exception of AIMS2-SF work data, which was only available for participants who were working, all missing data were imputed via multiple imputation using chained equations.^42^ Data was defined as missing if patients failed to complete either the 16-week or the 32-week follow-up. Nine per cent of responders dropped out of the study at week 16 and 20% dropped out at week 32, therefore 20 imputed data sets were computed on guidance that at a minimum, the number needed should approximate the percentage of incomplete cases.^42^ Age, gender, treatment group and baseline PKQ, ASES-Pain and ASES-Other symptoms scores and AIMS2-SF symptoms, affect, physical and social scores were included in the imputation model. All analyses were then undertaken on an intention-to-treat basis except for the AIMS2-SF work data, which followed a complete case analysis approach. A conservative complete case analysis of all data was also undertaken to assess the effect of the missing data. Summary statistics are presented as means and 95% CIs for continuous variables and numbers and percentages for categories. Differences between treatment groups were investigated using χ^2^ test (Fisher's exact test if the expected cell counts were less than five) or t test for categorical and continuous variables, respectively. Repeated measures analysis of variance was used to look for trends over time. All analyses were adjusted for baseline values using analysis of covariance. Bonferroni correction was applied to the p values for the primary outcomes ASES-Pain and ASES-Other symptoms in order to avoid type I errors due to multiple testing.^43^ Thus for the primary outcome measures p<0.025 was regarded as statistically significant and p<0.05 was regarded as statistically significant for all other tests. STATA V.12^44^ was used for all analyses.

## Results

### Participant characteristics and baseline scores by treatment group

**Figure 1:**
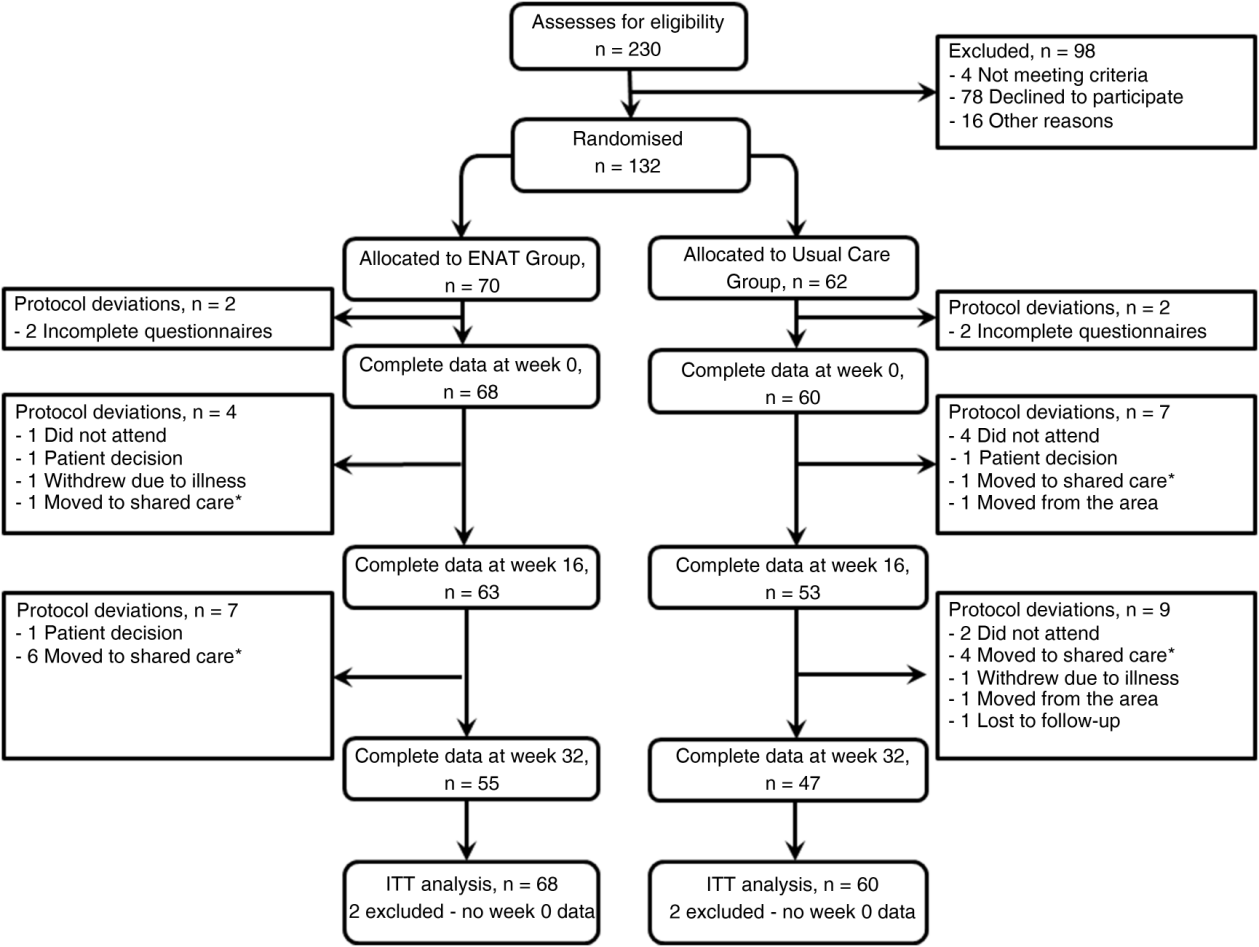
Study flow chart/*shared care, Clinical Nurse Specialist care was transferred to the primary care by the hospital.

A total of 132 patients were entered into the study (CG, 62 and IG, 70). Of the 132 patients who entered the study; 128 (97%) completed the questionnaires at baseline (60 (97%) CG, 68 (97%) IG), 116 (88%) at week 16 (53 (85%) CG, 63 (90%) IG) and 102 (77%) at week 32 (47 (76%) CG, 55 (79%) IG). Figure 1 presents the patient flow chart and table 1 presents the patient characteristics (and baseline values of outcome measures) by treatment group.

**Table 1:** Baseline characteristics of study population by treatment group

Patient characteristics and outcomes	Control group (N=60)	Intervention group (N=68)
Patient characteristics		
Mean age in years (SD)	56 (13.3)	54 (12.3)
Male (%)	22 (37%)	22 (32%)
Disease duration in years (SD)	6.7 (8.9)	5.2 (4.9)
Left school after 16 years of age (%)	20 (32%)	15 (21%)
Studied since leaving school (%)	29 (48%)	25 (37%)

**Outcomes**	**Mean (SD)**	**Mean (SD)**

ASES (score range)		
ASES-Pain (5–50)	25 (11.2)	23 (9.1)
ASES-Other symptoms (6–60)	34 (12.7)	31 (11.5)
AIMS2-SF (score range)		
AIMS2-SF: physical (0–10)	3.1 (2.59)	3.1 (2.03)
AIMS2-SF: symptoms (0–10)	5.0 (3.23)	5.4 (2.64)
AIMS2-SF: affect (0–10)	3.7 (2.13)	4.3 (2.19)
AIMS2-SF: social (0–10)	5.5 (1.73)	5.6 (1.75)
AIMS2-SF: work* (0–10)	2.0 (2.69)	2.3 (3.08)
ENAT† (score range)‡		
Managing pain (0–18)	–	13.3 (3.68)
Movement (0–15)	–	11.0 (2.88)
Feelings (0–12)	–	7.7 (3.22)
Arthritis process (0–21)	–	16.0 (3.90)
Treatments (0–21)	–	15.4 (4.43)
Self-help measures (0–18)	–	12.6 (4.10)
Support (0–12)	–	7.9 (2.65)

*Scores are only available for the 59 (26, 33) patients who were working.

†ENAT was used in the intervention group only.

‡ENAT scores are Rasch-transformed values.

AIMS2-SF, short-form of arthritis impact measurement scale (zero=good health status); ASES, Arthritis Self Efficacy Scale (higher score=greater self-efficacy); ENAT, Educational Needs Assessment Tool (zero=no need).

### Group differences in the interventions

The interventions undertaken by both groups are summarised in the online supplementary table S1. At week 16, there were no significant differences between the two groups in the number of interventions. At week 32, patients under CG were more likely to have medication or dosage changes than IG. Predictably, more patients under CG received patient education on drug therapy than those in the ENAT group. There were no significant differences between the two groups in the mean consultation times and number of referrals to other healthcare workers.

### Patient outcomes in the follow-up period

Table 2 presents the between-group differences after adjusting for baseline values using analysis of covariance in self-efficacy (ASES) and health status (AIMS2-SF) outcomes at week 16 and week 32. Table 3 presents the mean score changes over time for self-efficacy, health status and patients’ knowledge of their RA over the whole follow-up period (results of repeated measure analysis of variance).

**Table 2:** Follow-up data for self-efficacy and health status and between-group differences after adjusting for baseline values using analysis of covariance (results of intention-to-treat analyses)

	Control group (N=60)	Intervention group (N=68)	Mean difference	
Domain—time point (range)	Mean (95% CI)	Mean (95% CI)	ENAT-usual care (95% CI)	T-statistic (p value)
ASES (score range; higher score=greater self-efficacy)				
ASES-Pain—week 16 (5–50)	25.8 (23.8 to 27.8)	27.7 (26.2 to 29.2)	1.86 (−0.63 to 4.35)	1.48 (0.142)*
ASES-Pain —week 32 (5–50)	26.9 (25.2 to 28.5)	31.2 (30.0 to 32.5)	4.36 (1.17 to 7.55)	2.72 (0.008) *
ASES-Other symptoms—week 16 (6–60)	33.5 (31.6 to 35.5)	35.8 (34.2 to 37.3)	2.22 (−1.15 to 5.59)	1.31 (0.194)*
ASES-Other symptoms—week 32 (6–60)	34.0 (32.4 to 35.7)	39.9 (38.6 to 41.1)	5.84 (2.07 to 9.62)	3.07 (0.003)*
AIMS2-SF (score range; zero=good health status)				
AIMS2-SF: physical—week 16 (0–10)	3.01 (2.56 to 3.47)	2.81 (2.47 to 3.15)	−0.21 (−0.72 to 0.30)	−0.83 (0.409)
AIMS2-SF: physical—week 32 (0–10)	3.05 (2.56 to 3.53)	2.84 (2.48 to 3.20)	−0.21 (−0.76 to 0.34)	−0.77 (0.442)
AIMS2-SF: symptoms—week 16 (0–10)	4.77 (4.16 to 5.38)	4.56 (4.09 to 5.03)	−0.21 (−0.93 to 0.52)	−0.57 (0.572)
AIMS2-SF: symptoms—week 32 (0–10)	5.25 (4.76 to 5.74)	4.21 (3.79 to 4.63)	−1.04 (−1.85 to −0.22)	−5.54 (0.013)
AIMS2-SF: affect—week 16 (0–10)	4.00 (3.59 to 4.41)	3.84 (3.44 to 4.24)	−0.15 (−0.58 to 0.27)	−0.71 (0.479)
AIMS2-SF: affect—week 32 (0–10)	4.29 (3.94 to 4.64)	3.44 (3.10 to 3.78)	−0.86 (−1.46 to −0.26)	−2.84 (0.006)
AIMS2-SF: social week 16 (0–10)	5.60 (5.22 to 5.98)	5.45 (5.09 to 5.81)	−0.15 (−0.51 to 0.21)	−0.82 (0.414)
AIMS2-SF: social week 32 (0–10)	5.79 (5.44 to 6.14)	5.67 (5.34 to 6.00)	−0.12 (−0.61 to 0.38)	−0.47 (0.638)
AIMS2-SF: work†—week 16 (0–10)	3.47 (2.90 to 4.04)	2.70 (2.12 to 3.28)	−0.77 (−2.28 to 0.74)	−1.02 (0.311)
AIMS2-SF: work†—week 32 (0–10)	2.44 (1.99 to 2.89)	2.71 (2.25 to 3.17)	0.27 (−2.09 to 2.64)	0.24 (0.810)

AIMS2-SF, short form of arthritis impact measurement scale; ASES, Arthritis Self Efficacy Scale; ENAT, Educational Needs Assessment Tool.

*Bonferroni-adjusted p value=0.025 for significance at the α level.

†All missing data imputed except for AIMS2-SF: work where scores are only available for the patients who were working, week 16 and week 32 usual care N=26, intervention N=33.

**Table 3:** Mean score changes (95% CI) in the outcome measures over time*

	Week 0	Week 16	Week 32	
Control group (N=60)	Mean (95% CI)	Mean (95% CI)	Mean (95% CI)	F_2,118_ (p value)
ASES (score range; higher score=greater self-efficacy)				
ASES-Pain (5–50)	25.3 (22.4 to 28.2)	26.5 (23.9 to 29.2)	27.5 (24.6 to 30.3)	2.03 (0.135)†
ASES-Other symptoms (6–60)	34.2 (30.9 to 37.4)	35.0 (31.4 to 38.6)	35.3 (31.5 to 39.0)	0.40 (0.673)†
AIMS2-SF (score range; zero=good health status)				
AIMS2: physical (0–10)	3.1 (2.4 to 3.8)	3.0 (2.4 to 3.6)	3.0 (2.4 to 3.7)	0.09 (0.917)
AIMS2: symptoms (0–10)	5.0 (4.2 to 5.8)	4.6 (3.8 to 5.4)	5.1 (4.2 to 6.0)	2.34 (0.101)
AIMS2: affect(0–10)	3.7 (3.2 to 4.3)	3.8 (3.3 to 4.2)	4.1 (3.5 to 4.7)	2.18 (0.117)
AIMS2: social(0–10)	5.5 (5.1 to 6.0)	5.6 (5.1 to 6.0)	5.7 (5.3 to 6.2)	1.86 (0.160)
AIMS2: work‡(0–10)	2.0 (0.9 to 3.1)	3.4 (1.9 to 4.8)	2.4 (−0.01 to 4.8)	3.84 (0.028)
PKQ (score range, higher score=higher level of knowledge)				
Total PKQ (0–12)	8.6 (7.9 to 9.2)	8.7 (8.1 to 9.3)	9.1 (8.4 to 9.8)	1.96 (0.146)


**Intervention group (N=68)**	**Mean (95% CI)**	**Mean (95% CI)**	**Mean (95% CI)**	**F_2,134_ (p value)**

ASES(score range; higher score=greater self-efficacy)				
ASES-Pain (5–50)	23.4 (21.2 to 25.6)	27.0 (24.6 to 29.4)	30.7 (28.2 to 33.2)	37.5 (<0.001)†
ASES-Other symptoms (6–60)	30.6 (27.8 to 33.4)	34.5 (31.4 to 37.5)	38.7 (35.7 to 41.7)	37.2 (<0.001)†
AIMS2-SF (score range; zero=good health status)				
AIMS2: physical (0–10)	3.1 (2.6 to 3.6)	2.8 (2.3 to 3.3)	2.8 (2.3 to 3.4)	2.35 (0.099)
AIMS2: symptoms (0–10)	5.4 (4.8 to 6.1)	4.7 (4.0 to 5.4)	4.3 (3.7 to 5.0)	11.8 (<0.001)
AIMS2: affect (0–10)	4.3 (3.8 to 4.8)	4.1 (3.5 to 4.6)	3.6 (3.1 to 4.1)	12.0 (<0.001)
AIMS2: social (0–10)	5.6 (5.2 to 6.0)	5.5 (5.0 to 6.0)	5.7 (5.2 to 6.2)	1.35 (0.263)
AIMS2: work‡(0–10)	2.3 (1.2 to 3.4)	2.8 (1.6 to 3.9)	2.8 (1.5 to 5.0)	0.60 (0.552)
PKQ (score range, higher score=higher level of knowledge)				
Total PKQ (0–12)	8.6 (8.0 to 9.1)	9.0 (8.5 to 9.5)	9.2 (8.7 to 9.8)	5.22 (0.007)

*Analysis not adjusted for baseline values as examining changes over time including values at baseline.

†Bonferroni-adjusted p value=0.025 for significance at the α level.

‡All missing data imputed except for AIMS2-SF: Work where scores are only available for the patients who were working, week 16 and week 32 control group N=26 and intervention group N=33.

ASES, Arthritis Self Efficacy Scale; PKQ, patient knowledge questionnaire.

#### Self-efficacy

At week 16 there were no significant between-group differences in the ASES scores. At week 32 however, mean scores for ASES-Pain and ASES-Other symptoms were higher for IG than CG; ASES-Pain mean difference=4.36, 95% CI 1.17 to 7.55; t=2.72, p=0.008; ASES-Other symptoms mean difference=5.84, 95% CI 2.07 to 9.62; t=3.07, p=0.003 (Bonferroni-adjusted p value=0.025 for significance at the α level). Over the whole follow-up period, there were no significant changes in ASES scores in CG but IG saw significant improvements in ASES-Pain and ASES-Other symptoms scores (table 3).

### Health status

While there were no significant between-group differences at week 16, there were significant between-group differences in favour of IG at week 32 in AIMS2-SF Symptoms and AIMS2-SF Affect. There were no significant differences in other AIMS2-SF scores. See table 2. Over the whole follow-up period, IG saw significant decrease in AIMS2-SF scores for symptoms and affect domains, CG had improvements in the work domain only. See table 3.

#### Patients’ knowledge

Online supplementary table S2 presents the number of patients providing correct answers to each PKQ item and the mean PKQ score (95% CI) at each time point. There were no significant between-group differences in the number of patients giving the correct responses to PKQ items or mean PKQ scores at any time point. Over the follow-up period, the patients in both groups revealed a trend of increase in their total PKQ scores, but this was only significant in the ENAT group. See table 3.

### Educational needs (ENAT scores) of patients in IG

The first screening question on ENAT asks patients if they want any education about their arthritis (yes/no response). At week 0, 33 patients (48%) responded with a ‘yes’ and this dropped to 13 (21%) at week 16 and 9 (16%) by week 32 (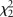
=18.76, p<0.001). Table 4 presents ENAT scores over time. There was a change in the amount of knowledge patients wanted over time with less people wanting to know everything by week 32, the change was statistically significant, though not at week 16 (week 0 vs week 16 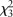
=5.12, p=0.163; week 0 vs week 32
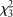
=8.06, p=0.045). The ENAT domain scores dropped between week 0 and week 16. Repeated measures analysis revealed significant decrease in all ENAT domain scores (except feelings) and the total ENAT score.

**Table 4:** Change in educational needs assessment tool (ENAT) scores* over time

	Week 0 (N=68)	Week 16 (N=68)	Week 32 (N=68)	
Initial question of need	**N (%)**	**N (%)**	**N (%)**	
Do not want to know anything	2 (3)	3 (4)	5 (7)	
Want to know some things	15 (22)	24 (35)	25 (37)	
Want to know lots of things	10 (15)	13 (19)	13 (19)	
Want to know everything	41 (60)	28 (41)	25 (37)	

ENAT domains (score range)*	Mean*(95% CI)	Mean* (95% CI)	Mean* (95% CI)	**F_2,107_ (p value)**

Managing pain (0–18)	13.3 (12.4 to 14.2)	12.2 (11.1 to 13.3)	11.5 (10.5 to 12.5)	8.07 (0.001)
Movement (0–15)	11.0 (10.3 to 11.7)	10.0 (9.1 to 10.9)	9.9 (9.1 to 10.7)	3.16 (0.046)
Feelings (0–12)	7.7 (6.9 to 8.5)	6.9 (6.0 to 7.9)	6.9 (6.1 to 7.7)	2.09 (0.129)
Arthritis process (0–21)	16.0 (15.1 to 17.0)	14.2 (13.0 to 15.3)	14.2 (13.0 to 15.4)	6.23 (0.003)
Treatments (0–21)	15.4 (14.3 to 16.5)	14.5 (13.4 to 15.5)	13.7 (12.6 to 14.8)	3.46 (0.035)
Self-help measures (0–18)	12.6 (11.6 to 13.6)	11.3 (10.2 to 12.4)	10.7 (9.6 to 11.9)	8.02 (0.001)
Support (0–12)	7.9 (7.3 to 8.5)	7.3 (6.5 to 8.1)	6.5 (5.8 to 7.3)	5.93 (0.004)
Total ENAT score (0–117)	83.9 (79.2 to 88.6)	76.3 (70.4 to 82.3)	73.8 (67.8 to 79.7)	7.53 (0.001)

*The ENAT domain scores are Rasch-transformed values, see scoring guide in the online supplementary material.

## Discussion

While ENAT has been validated in several countries across Europe^31–33^ and it has been used in research as an outcome measure, this was the first RCT to assess its effects on patients’ outcomes. This pragmatic study has demonstrated the effects of using ENAT as a template from which to give need-based patient education in normal clinical settings. In this study, although both groups were provided with patient education by an experienced CNS and had the same consultation duration, IG saw significant improvements in self-efficacy and some aspects of health status compared with CG at 32 weeks follow-up. This suggests that the needs-based education adds value to this provision by improving self-efficacy and by ensuring that patients’ priority educational needs are being met.

Self-efficacy is important for people with arthritis who are expected to undertake self-management activities and adhere to different therapeutic interventions in addition to their family roles and work. In chronic disease, self-efficacy has been shown to mediate the effects of education onto other outcomes such as pain, physical health status and mental health status and health-related quality of life.^9^ ^11^ ^12^ This is likely to explain the improvement in the AIMS2-SF symptoms and AIMS2-SF affect that was seen in IG. In a 5-year observational study, Brekke *et al*^9^ found significant correlations between changes in self-efficacy and changes in AIMS2-Symptoms and AIMS2-Affect but not the AIMS2-Physical or AIMS2-Social subscales. It is perhaps not surprising that our 6-month study did not show improvements in these subscales.

The results on overall patient knowledge were interesting as there were no significant between-group differences in the mean PKQ scores at any time point. However, the PKQ scores within IG increased significantly, coupled with a decrease in their ENAT scores, which suggests that patients’ specific educational needs were being met. It is worth pointing out here that ENAT is meant to assess patients’ educational needs, not knowledge, therefore the use of ENAT may not result in an increase in the overall knowledge about RA. It is hoped that the clinician will use their own assessment skills in addition to ENAT as a simple tool to objectively address areas that are of greatest need from the patients’ point of view. Once the clinician has addressed patient's priority educational needs, other important aspects of patient education can be introduced such as need for switching drugs or cardiovascular risks.

Qualitative study of patients’ views and clinical usability of ENAT has demonstrated patients’ and nurses’ acceptability of the tool.^45^ Patients’ completion of ENAT enables them to think of questions which they would not have otherwise considered, this helps them to objectively and effectively identify their own educational needs and helps the clinicians to target areas that are often overlooked but important to patients such as sexual activity and support groups. This needs-based approach is likely to ensure that patients’ educational needs are being met effectively and that they are enabled to self-manage their disease and maximise their coping strategies.

This study had four main strengths. First, being a pragmatic RCT implies that the effects of ENAT demonstrated in this study have taken into account everyday clinical practice, meaning that it is feasible to use ENAT in normal clinical settings and expect similar results. Second, being a multicentre study, the results are applicable to more than just one centre. Third, being a single-blind (participants) RCT helped reduce biased responses. Lastly, undertaking an intention-to-treat analysis with multiple imputations for the missing values, helped to reduce bias in the assessment of treatment effects. The exception to this approach was the AIMS2-SF work data which was not imputed.

This study had four main limitations. First, the follow-up period was only 6 months and since RA is a variable disease a longer follow-up period would have been desirable in order to reflect the long-term effects of needs-based education. However, it is expected that changes in self-efficacy especially early in the disease may contribute towards long-term effects in health outcomes.^9^ Second, although the between-group differences in both ASES subscales were highly significant at week 32, the prehypothesised difference^36^ of 5.5 was not reached in the ASES-Pain subscale and we do not know if the associated changes in the AIMS2-SF subscales are large enough to be clinically significant. Third, the effectiveness of individual patient education may be influenced by how CNS relate to their patients. Since we did not measure this factor, and given that both interventions were provided by the same CNS, there may be a potential risk of performance bias by the intervention providers. Lastly, since the practitioners participating in this study were all CNS running nurse-led clinics, we do not know if ENAT would have had the same effects in rheumatologist-led clinics. Since rheumatologists’ clinics are characterised by problem-based interaction style and provision of factual biomedical information,^46^ ^47^ it is plausible to expect at least the same results from the use of ENAT in their consultations.

In conclusion, since targeted education is recommended as an integral part of management of RA,^6^ ^8^ ENAT is a worthy tool which should be given serious consideration as a template upon which to guide individualised patient education. The results of this RCT suggest that need-based education helps improve patients’ self-efficacy and some aspects of health status. Further research is required to determine the time in the RA trajectory where focused, patient-centred education has its maximum effects. Future developments of ENAT will ensure that the tool remains current and useful in connecting patients to available resources and services.

## Supplementary Material

Web supplement

Web tables
