# Supplementary material for: Effects of needs-based patient education on self-efficacy and health outcomes in people with rheumatoid arthritis: a multicentre, single blind, randomised controlled trial
Source: Ann Rheum Dis. 2015 Jul 10;75(6):1126–32. doi: 10.1136/annrheumdis-2014-207171 (PMC4893097; doi:10.1136/annrheumdis-2014-207171)
Supplement: Web supplement [file annrheumdis-2014-207171-s1.pdf]

The Educational Needs Assessment Tool with 4-point rating scales

(The ENAT-2)

Background information

The Educational Needs Assessment Tool is a self-administered questionnaire containing 39 statements which measure the educational needs of patients with rheumatic diseases. The statements are grouped into 7 domains that measure specific aspects of 'educational needs'. These items are:

1. Managing pain
2. Movement
3. Feelings
4. Arthritis process
5. Treatment form health professionals
6. Self-help measures
7. Support from others

Uses of the ENAT

The ENAT can be used by clinicians in wards or clinics to assess what are the most important educational / informational needs from the patient's point of view. This information, along with the clinicians' perception of what the patient needs to know, will allow the provision of timely and meaningful education tailored to the needs of each individual patient.

The ENAT can also be used by researchers to assess the educational needs of patients at particular time points. For example, changes in educational needs could be assessed by comparing results from the ENAT at the start and finish of a study.

For use in audit or in research (where measurement aspect is important), the ENAT-2 needs to be coded and scored using the following steps:

(i) The ENAT-2 rating scales descriptives should be number-coded thus:

Not at all important = 0
A little important = 1
Very important = 2
Extremely important = 3

See example 1:

Table 1: This item relates to your Feelings:

**How important is it for you NOW to know more about the following:**	Not at all important **(0)**	A little important **(1)**	Very Important **(2)**	Extremely important **(3)**
Ways to deal with stress	√			
Ways to deal with moods or depression				√
Why I am feeling tired			√	
Why I am feeling down or depressed				√

(ii) Following the coding, the scores for each statement are then added up to provide the **raw domain score**. In the example above, the domain score = 8 (the domain score for feelings should range between 0 – 12).

(iii) The raw domain scores obtained in (ii) can be transformed into linear scale to enable their use in parametric analyses. This is done by using the conversion table provided below. In example 1 for feelings, the raw domain score of 8, corresponds to **transformed domain score** of 7.6 (See table 1).

(iv) Adding up all the transformed domain scores gives the **total ENAT score** which is an estimate of the patient's educational needs (range = 0 -117).

Table 1: Conversion of raw Domain scores into Rasch-transformed domain scores

**Raw scores**	**Rasch transformed (interval level) scores**						
	Pain	Movement	Feelings	Arthritis	Treatments	Self-Help	Support
**0**	0.0	0.0	0.0	0.0	0.0	0.0	0.0
**1**	1.6	2.1	1.5	1.3	2.0	1.0	1.4
**2**	2.7	3.6	2.6	2.4	3.4	1.9	2.5
**3**	3.7	4.9	3.5	3.3	4.5	2.6	3.5
**4**	4.5	5.9	4.4	4.0	5.4	3.3	4.5
**5**	5.3	6.9	5.1	4.7	6.3	3.9	5.7
**6**	6.2	7.9	6.0	5.5	7.2	4.6	7.2
**7**	7.3	8.9	6.8	6.2	8.1	5.6	8.3
**8**	8.6	9.6	7.6	7.2	9.0	6.8	9.0
**9**	10.0	10.3	8.5	8.3	10.1	8.5	9.7
**10**	11.4	10.9	9.4	9.6	11.1	10.3	10.3
**11**	12.4	11.5	10.6	11.0	12.2	11.7	11.1
**12**	13.2	12.2	12.0	12.3	13.2	12.7	12.0
**13**	13.9	12.8		13.5	14.1	13.5	
**14**	14.5	13.8		14.5	14.8	14.2	
**15**	15.2	15.0		15.2	15.5	15.0	
**16**	15.9			16.0	16.2	15.8	
**17**	16.8			16.7	16.9	16.8	
**18**	18.0			17.5	17.6	18.0	
**19**				18.3	18.4		
**20**				19.5	19.5		
**21**				21.0	21.0		

The ENAT can be obtained from the University of Leeds Psychometric Laboratory via the url below:
http://www.leeds.ac.uk/medicine/rehabmed/psychometric/Scales1.htm.

The ENAT can be cited using the following references

1. Hardware B, Anne Lacey E, Shewan J. Towards the development of a tool to assess educational needs in patients with arthritis. Clin Eff Nurs 2004;**8**(2):111-17.
2. Ndosi M, Tennant A, Bergsten U, et al. Cross-cultural validation of the Educational Needs Assessment Tool in RA in 7 European countries. BMC Musculoskelet Disord 2011;**12**:110.
3. Ndosi M, Bremander A, Hamnes B, et al. Validation of the educational needs assessment tool as a generic instrument for rheumatic diseases in seven European countries. Ann Rheum Dis 2014;**73**(12):2122-9.

Prepared by Dr Mwidimi Ndosi, July 2013
