# Supplementary material for: Effects of needs-based patient education on self-efficacy and health outcomes in people with rheumatoid arthritis: a multicentre, single blind, randomised controlled trial
Source: Ann Rheum Dis. 2015 Jul 10;75(6):1126–32. doi: 10.1136/annrheumdis-2014-207171 (PMC4893097; doi:10.1136/annrheumdis-2014-207171)
Supplement: Web tables [file annrheumdis-2014-207171-s2.pdf]

Supplementary table S1: The CNS consultation checklist over time (weeks 0, 16 & 32) by treatment group

Interventions	Control Group	Intervention Group		Control Group	Intervention Group		Control Group	Intervention Group	
		Week 0			Week 16			Week 32	
Interventions	(N = 60)	(N = 68)	χ_1^2 (p-value)	(N = 60)	(N = 68)	χ_1^2 (p-value)	(N = 60)	(N = 68)	χ_1^2 (p-value)*
Change patients medication/dose	14 (23%)	29 (43%)	5.33 (0.021)	14 (23%)	16 (24%)	0.001 (0.979)	13 (28%)	6 (11%)	4.69 (0.030)
Prescribed Intra-articular steroid injection	0 (0.0%)	2 (2.9%)	0.498*	0 (0%)	0 (0%)	N/A	3 (6%)	3 (6%)	1.000*
Prescribed IM steroid injection	1 (1.7%)	2 (2.9%)	1.000*	2 (3%)	2 (3%)	1.000*	0 (0.0%)	0 (0.0%)	N/A
Conferred with rheumatologist	8 (13%)	13 (19%)	0.78 (0.378)	12 (20%)	9 (13%)	1.06 (0.302)	8 (17%)	7 (13%)	0.37 (0.542)
Referred to physiotherapist	1 (1.7%)	6 (8.8%)	0.120*	1 (2%)	2 (3%)	1.000*	0 (0.0%)	2 (4%)	0.498*
Referred to occupational therapist	3 (5.0%)	13 (19%)	5.81 (0.016)	1 (2%)	1 (1%)	1.000*	0 (0.0%)	3 (6%)	0.247*
Referred to podiatrist	1 (1.7%)	2 (2.9%)	1.000*	2 (3%)	1 (1%)	0.600*	0 (0.0%)	1 (2%)	1.000*
Referred to dietician	0 (0.0%)	0 (0.0%)	N/A	0 (0%)	0 (0%)	N/A	0 (0.0%)	0 (0.0%)	N/A
Referred to social worker	0 (0.0%)	0 (0.0%)	N/A	0 (0%)	0 (0%)	N/A	0 (0.0%)	0 (0.0%)	N/A
Referred to appliance officer	0 (0.0%)	0 (0.0%)	N/A	0 (0%)	1 (1%)	1.000*	1 (2%)	1 (2%)	1.000*
Referred to orthopaedic surgeon	0 (0.0%)	0 (0.0%)	N/A	0 (0%)	0 (0%)	N/A	1 (2%)	0 (0.0%)	0.469*
Referred to other consultant	0 (0.0%)	0 (0.0%)	N/A	1 (2%)	0 (0%)	0.469*	0 (0.0%)	1 (2%)	1.000*
Referred to other MDT member	0 (0.0%)	0 (0.0%)	N/A	1 (2%)	1 (1%)	1.000*	0 (0.0%)	1 (2%)	1.000*
Referred for admission to rheumatology ward	0 (0.0%)	0 (0.0%)	N/A	0 (0%)	0 (0%)	N/A	0 (0.0%)	2 (4%)	0.498*
Provide education on: causes of RA	45 (75%)	51 (75%)	0.00 (0.999)	28 (53%)	34 (54%)	0.02 (0.903)	23 (49%)	29 (53%)	0.15 (0.703)
Provide education on: drug therapy	53 (88%)	65 (96%)	2.33 (0.127)	48 (91%)	56 (89%)	0.09 (0.768)	45 (96%)	44 (80%)	8.77 (0.012)
Provide education on: managing pain	47 (78%)	60 (88%)	2.28 (0.131)	42 (79%)	51 (81%)	0.05 (0.818)	37 (79%)	41 (75%)	0.25 (0.620)
Provide education on: managing stiffness	33 (55%)	36 (55%)	0.05 (0.816)	33 (62%)	40 (63%)	0.02 (0.892)	31 (66%)	30 (55%)	1.37 (0.241)
Provide education on: energy conservation	19 (32%)	25 (37%)	0.37 (0.545)	16 (30%)	24 (38%)	0.80 (0.372)	21 (45%)	21 (38%)	0.44 (0.506)
Provide education on: rest and sleep	40 (67%)	47 (69%)	0.09 (0.767)	34 (64%)	46 (73%)	1.06 (0.304)	32 (68%)	37 (67%)	0.01 (0.930)
Provide education on: managing fatigue	26 (43%)	34 (50%)	0.57 (0.451)	30 (57%)	36 (57%)	0.003 (0.953)	29 (62%)	26 (47%)	2.12 (0.145)
Provide education on: diet	24 (40%)	36 (53%)	2.14 (0.143)	17 (32%)	26 (41%)	1.04 (0.307)	26 (55%)	29 (53%)	0.07 (0.794)
Provide education on: use of splints	14 (23%)	24 (35%)	2.18 (0.139)	12 (23%)	16 (25%)	0.12 (0.730)	10 (21%)	16 (29%)	0.81 (0.367)

Provide education on: exercise	37 (62%)	46 (68%)	0.50 (0.479)	28 (53%)	40 (63%)	1.35 (0.245)	25 (53%)	31 (56%)	0.10 (0.748)
Provide education on: anxiety or depression	22 (37%)	34 (50%)	2.30 (0.129)	21 (40%)	30 (48%)	0.75 (0.387)	18 (38%)	21 (38%)	0.0001 (0.990)
Provide education on: effects on family	30 (50%)	42 (62%)	1.79 (0.181)	26 (49%)	33 (52%)	0.13 (0.721)	26 (55%)	27 (49%)	0.39 (0.530)
Provide education on: effects on work	23 (38%)	33 (49%)	1.35 (0.246)	21 (40%)	27 (43%)	0.12 (0.725)	21 (45%)	18 (33%)	1.53 (0.216)
Provide education on: sexual activity	2 (3%)	10 (15%)	4.85 (0.028)	1 (2%)	6 (10%)	0.120*	2 (4%)	6 (11%)	0.281*
Provide education on: tests for RA	22 (37%)	29 (43%)	0.48 (0.480)	20 (38%)	23 (37%)	0.02 (0.892)	23 (49%)	21 (38%)	1.19 (0.274)
Provide education on: complimentary therapy	11 (18%)	22 (32%)	4.23 (0.121)	10 (19%)	16 (25%)	0.71 (0.401)	8 (17%)	13 (24%)	0.68 (0.410)
Provide education on: written material	26 (43%)	35 (51%)	0.85 (0.358)	15 (28%)	18 (29%)	0.001 (0.974)	15 (32%)	16 (29%)	0.10 (0.757)
Provide education on: website	16 (27%)	24 (35%)	1.10 (0.293)	7 (13%)	7 (11%)	0.12 (0.730)	3 (6%)	10 (18%)	3.17 (0.075)
Provide education on: group patient education	9 (15%)	15 (22%)	1.04 (0.307)	7 (13%)	12 (19%)	0.72 (0.397)	7 (15%)	6 (11%)	0.36 (0.548)
Provide education on: support groups	11 (18%)	23 (34%)	3.92 (0.048)	10 (19%)	13 (21%)	0.06 (0.812)	6 (13%)	9 (16%)	0.26 (0.609)
Provide education on: other	1 (1.7%)	1 (1.5%)	1.000*	0 (0%)	3 (5%)	0.247*	2 (4%)	4 (7%)	0.684*
Provide psychosocial support	27 (45%)	34 (50%)	0.32 (0.572)	23 (43%)	32 (51%)	0.63 (0.427)	24 (51%)	24 (44%)	0.56 (0.454)
Approximate consultation duration in mins (SD)	25.8 (9.62)	26.3 (8.49)		21.5 (4.76)	23.4 (8.29)		22.9 (9.48)	22.7 (8.98)	

*When expected cell counts are small (< 5) chi-squared test is not appropriate. Fishers' Exact test has been used when this has occurred and p-values are presented.

Supplementary Table S2: Correct responses to the PKQ by treatment group*

	Control group	Intervention group	Risk difference % (95% CI)	χ_1^2 (p-value)
**Week 0**	**N = 60**	**N = 68**		
What causes rheumatoid arthritis	30 (50%)	43 (63%)	13.2 (-3.8 to 30.3)	2.28 (0.131)
Symptoms of an acute flare of RA	48 (80%)	58 (85%)	5.3 (-7.9 to 18.5)	0.63 (0.428)
Why do you take 2nd line medication	49 (82%)	57 (84%)	2.2 (-11.0 to 15.3)	0.10 (0.747)
How does a non-steroid anti-inflammatory work	47 (78%)	52 (76%)	-1.9 (-16.4 to 12.6)	0.06 (0.802)
What would you do if you developed a rash	54 (90%)	57 (84%)	-6.2 (-17.8 to 5.4)	1.06 (0.304)
Why is regular blood monitoring important	56 (93%)	62 (91%)	-2.2 (-11.4 to 7.1)	0.21 (0.650)
Which 2 blood tests indicate how active your arthritis is	21(35%)	26 (38%)	3.2 (-13.5 to 19.9)	0.14 (0.705)
When would you use an ice pack	47 (78%)	54(79%)	1.1 (-13.1 to 15.5)	0.02(0.881)
How often should you do a home exercise programme	35 (58%)	46 (68%)	9.3 (-7.4 to 26.0)	1.19 (0.275)
If you had a hot painful knee would you …	41 (68%)	43 (63%)	-5.1 (-23.8 to 11.3)	0.37 (0.545)
Which of the following is an example of energy conservation	42 (70%)	39 (57%)	-12.6 (-29.2 to 3.9)	2.19 (0.139)
Which of the following is the best routine	55 (92%)	57 (84%)	-7.8 (-19.0 to 3.4)	1.79 (0.181)
Mean PKQ score (95% CI) - score range 0-12	8.8 (8.2 to 9.3)	8.7 (8.2 to 9.2)	-0.06 (-0.81 to 0.69)	t=-0.16 (0.871)
**Week 16**	**N = 60**	**N = 68**		
What causes rheumatoid arthritis	38 (63%)	43 (63%)	1.0 (-16.8 to 16.6)	0.00 (0.991)
Symptoms of an acute flare of RA	49 (82%)	57 (84%)	3.8 (-19.2 to 26.7)	0.10 (0.747)
Why do you take 2nd line medication	46 (77%)	56 (82%)	8.7 (-12.7 to 30.2)	0.64 (0.425)
How does a non-steroid anti-inflammatory work	50 (83%)	56 (82%)	-1.7 (-24.6 to 21.2)	0.02 (0.883)
What would you do if you developed a rash	53 (88%)	60 (88%)	-0.1 (-11.3 to 11.1)	0.00 (0.986)
Why is regular blood monitoring important	54 (90%)	62 (91%)	1.2 (-9.0 to 11.3)	0.05 (0.820)
Which 2 blood tests indicate how active your arthritis is	25 (42%)	33 (49%)	6.9 (-10.4 to 24.1)	0.61 (0.436)
When would you use an ice pack	47 (78%)	56 (82%)	4.0 (-9.8 to 17.8)	0.33(0.567)
How often should you do a home exercise programme	39 (65%)	49 (72%)	7.1 (-9.0 to 23.2)	0.74 (0.390)
If you had a hot painful knee would you …	38 (63%)	44 (65%)	1.4 (-15.3 to 18.0)	0.03 (0.872)
Which of the following is an example of energy conservation	33 (55%)	37 (54%)	-0.6 (-17.9 to 16.7)	0.00 (0.947)
Which of the following is the best routine	55 (92%)	57 (84%)	-7.8 (-19.0 to 3.4)	1.79 (0.181)
Mean PKQ score (95% CI) - score range 0-12	8.8 (8.2 to 9.4)	9.1 (8.5 to 9.6)	0.3 (-0.5 to 1.1)	t=0.74(0.460)
**Week 32**	**N = 60**	**N = 68**		

What causes rheumatoid arthritis	45 (75%)	45 (66%)	-8.8 (-24.5 to 6.9)	1.19 (0.276)
Symptoms of an acute flare of RA	50 (83%)	53 (78%)	-5.4 (-19.0 to 8.2)	0.59 (0.443)
Why do you take 2nd line medication	53 (88%)	57 (84%)	-4.5 (-16.5 to 7.4)	0.54 (0.464)
How does a non-steroid anti-inflammatory work	51 (85%)	60 (88%)	3.2 (-8.6 to 15.1)	0.29 (0.590)
What would you do if you developed a rash	50 (83%)	62 (91%)	7.8 (-3.7 to 19.4)	1.79 (0.181)
Why is regular blood monitoring important	56 (93%)	63 (93%)	-0.7 (-9.5 to 8.2)	0.02 (0.880)
Which 2 blood tests indicate how active your arthritis is	31 (52%)	30 (44%)	-7.5 (-24.8 to 9.7)	0.73 (0.394)
When would you use an ice pack	48 (80%)	59 (84%)	4.3 (-8.9 to 17.5)	0.41(0.523)
How often should you do a home exercise programme	42 (70%)	49 (72%)	2.1 (-13.7 to 17.8)	0.07 (0.798)
If you had a hot painful knee would you …	45 (75%)	51 (74%)	-1.1 (-16.2 to 14.0)	0.02 (0.888)
Which of the following is an example of energy conservation	36 (60%)	41 (60%)	0.3 (-16.7 to 17.3)	0.00 (0.973)
Which of the following is the best routine	54 (90%)	58 (85%)	-4.7 (-16.0 to 6.6)	0.65 (0.422)
Mean PKQ score (95% CI) - score range 0-12	9.5 (8.9 to 10.1)	9.2 (8.6 to 9.9)	-0.29 (-1.14 to 0.56)	t=-0.66(0.508)

*The numbers in the table present those answering correctly for each question except the final row, which is the mean score for each group
